# Complete mitochondrial genome and the phylogenetic position of the Bowmouth guitarfish *Rhina ancylostoma* (Rajiformes, Rhinobatidae)

**DOI:** 10.1080/23802359.2016.1159935

**Published:** 2016-03-29

**Authors:** Ranran Si, Hao Chen, Weiming Ai, Xiao Chen, Shaobo Chen

**Affiliations:** a Department of Marine Science, Wenzhou Medical University, Wenzhou, Zhejiang, PR China;; b Zhejiang Mariculture Research Institute, Wenzhou, Zhejiang, PR China

**Keywords:** Rajiformes, *Rhina ancylostoma*, Rhinobatidae

## Abstract

In this study, the complete mitochondrial genome of the Bowmouth guitarfish *Rhina ancylostoma* (Rajiformes, Rhinobatidae) was first determined. The total length of this circle DNA was 17 217 bp, consisted of 37 genes with a typical gene order in vertebrate mitogenome. It had 42 bp short intergenic spaces and 40 bp overlaps. The nucleotide composition in *R. ancylostoma* was as follows: A, 33.0%; C, 25.1%; G, 12.6% and T, 29.3%. Two start codons (GTG and ATG) and two stop codons (TAG and TAA/T) were used in the protein-coding genes. The 22 tRNA genes were ranged from 67 bp (tRNA-*Ser2*) to 75 bp (tRNA-*Leu1*). The phylogenetic result showed that *R. ancylostoma* was clustered with the Rhinobatos.

The Bowmouth guitarfish *Rhina ancylostoma* (Rajiformes, Rhinobatidae), a widely distributed Indo-west Pacific inshore species, mainly occurs on or close to the seabed, which was a viviparous mammals (Carpenter et al. 1997; Compagno & Last 1999). It was very little known about the molecular and genetic research of *R. ancylostoma*. In this study, we first determined the complete mitochondrial genome of *R. ancylostoma* and analyzed the phylogenetic relationship in Rajiformes.

One specimen of *R. ancylostoma* (Museum of marine biology of Wenzhou Medical University, voucher XM2012032117) captured in the Taiwan Strait, was collected from a fish market in Xiamen, China. The experimental protocol and data analysis methods followed Chen et al. (2013). Including *R. ancylostoma*, 13 species of Rajiformes, with the complete mitogenomes available in the Genbank, were selected to construct the phylogenetic tree using the Bayesian method. The outgroup was *Dasyatis akajei* (Myliobatoformes).

The complete mitochondrial DNA of *R. ancylostoma* was 17 217 bp (Genbank accession no. KU721837), with a typical mitogenomic organization and gene order as most vertebrates, containing 13 protein-coding genes, 22 tRNA genes, 2 rRNA genes and 1 non-coding control region. The nucleotide composition in *R. ancylostoma* was as follows: A, 33.0%; C, 25.1%; G, 12.6% and T, 29.3%. The whole mitogenome had 42 bp short intergenic spaces located in 15 gene junctions and 40 bp overlaps located in seven gene junctions. Two start codons (GTG and ATG) and two stop codons (TAG and TAA/T) were used in the protein-coding genes, and most of them shared common initial codon ATG and terminal codon TAA/T. A non-standard initial codon GTG was owned by the *COI* gene, which was common in vertebrates (Slack et al. 2003). The *COII* and *ND4* genes were terminated with a single T, which could be extended to complete TAA through polyadenylation after transcriptions (Ojala et al. 1981). The 22 tRNA genes interspersed between the rRNAs and protein-coding genes, ranging from 67 bp (tRNA*-Ser2*) to 75 bp (tRNA*-Leu1*), while the 12S and 16S rRNA genes were located between the tRNA-*Phe* and tRNA-*Leu1* genes, separated by the tRNA-*Val* genes. A 39 bp inserted sequence was identified as the origin of light-strand replication (OL) between tRNA-*Asn* and tRNA-*Cys* genes with a stem-loop structure. The control region was 1499 bp, presenting a high A + T content (68.0%). There were a 48 bp motif repeated five times and a 46 bp motif repeated seven times in the control region. The high similarity (71.43%) suggested that they were homologous.

Within the Rajiformes, 13 available species belonged to three families with the (Rhinobatidae + (Rajidae + Arhynchobatidae)) relationship were consistent to the morphological result (McEachran & Dunn 1998). *Rhina ancylostoma* was sister to the Rhinobatos clade, then this clade clustered to *Zapteryx exasperata* (Figure 1).

**Figure 1.**
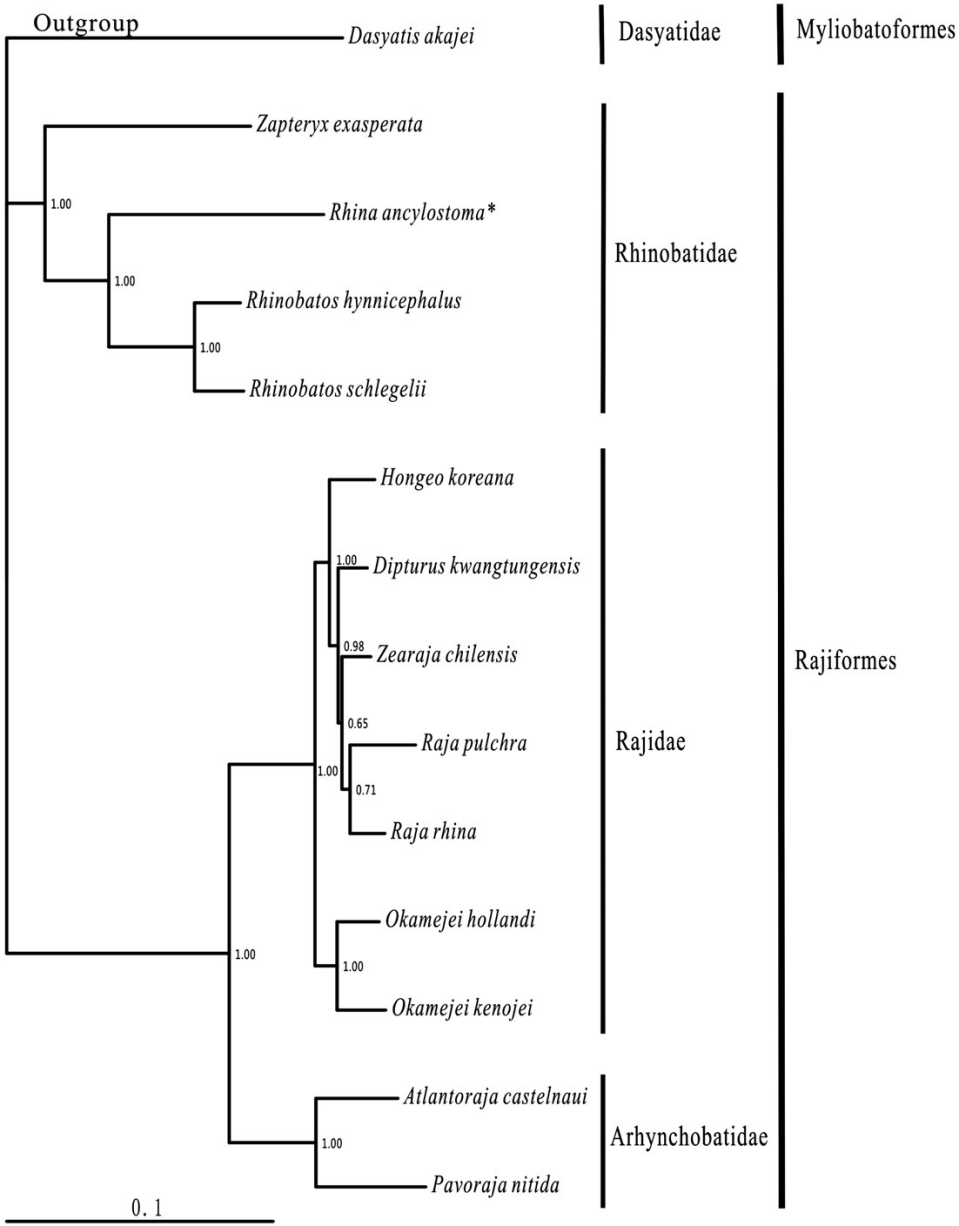
Phylogenetic position of *Rhina ancylostoma Dasyatis akajei* (NC_021132.1) was selected as the outgroup. The 13 species of Rajiformes were *Zapteryx exasperata* (NC_024937.1), *Rhina ancylostoma* (KU721837), *Rhinobatos hynnicephalus* (NC_022841.1), *R. schlegelii* (NC_023951.1), *Hongeo koreana* (NC_021963.1), *Dipturus kwangtungensis* (NC_023505.2), *Zearaja chilensis* (KJ913073.2), *Raja pulchra* (NC_025498.1), *Raja rhina* (KC914434.1), *Okamejei hollandi* (KP756687.1), *O. kenojei* (NC_007173.1), *Atlantoraja castelnaui* (NC_025942.1), and *Pavoraja nitida* (NC_024599.1).
